# Association of Amyloid and Tau With Cognition in Preclinical Alzheimer Disease: A Longitudinal Study

**DOI:** 10.1001/jamaneurol.2019.1424

**Published:** 2019-06-03

**Authors:** Bernard J. Hanseeuw, Rebecca A. Betensky, Heidi I. L. Jacobs, Aaron P. Schultz, Jorge Sepulcre, J. Alex Becker, Danielle M. Orozco Cosio, Michelle Farrell, Yakeel T. Quiroz, Elizabeth C. Mormino, Rachel F. Buckley, Kathryn V. Papp, Rebecca A. Amariglio, Ilse Dewachter, Adrian Ivanoiu, Willem Huijbers, Trey Hedden, Gad A. Marshall, Jasmeer P. Chhatwal, Dorene M. Rentz, Reisa A. Sperling, Keith Johnson

**Affiliations:** 1 Department of Radiology, Massachusetts General Hospital, the Gordon Center for Medical Imaging and the Athinoula A. Martinos Center for Biomedical Imaging, Boston; 2 Department of Neurology, Massachusetts General Hospital, Harvard Medical School, Boston; 3 Department of Neurology, Cliniques Universitaires Saint-Luc, Brussels, Belgium; 4 Department of Biostatistics, Harvard T. H. Chan School of Public Health, Boston, Massachusetts; 5 Faculty of Health, Medicine and Life Sciences, School for Mental Health and Neuroscience, Alzheimer Centre Limburg, Maastricht University, Maastricht, the Netherlands; 6 Department of Neurology and Neurological Sciences, Stanford University, California; 7 Center for Alzheimer Research and Treatment, Department of Neurology, Brigham and Women’s Hospital, Harvard Medical School, Boston, Massachusetts; 8 The Florey Institute, The University of Melbourne, Victoria, Australia; Melbourne School of Psychological Science, University of Melbourne, Victoria, Australia; 9 Dementia Research Group, BioMedical Research Institute, Hasselt University, Hasselt, Belgium; 10 Institute of Neuroscience, Université Catholique de Louvain, Brussels, Belgium; 11 Department of Cognitive Science and Artificial Intelligence, Tilburg University, Tilburg, the Netherlands

## Abstract

**Importance:**

Positron emission tomography (PET) imaging now allows in vivo visualization of both neuropathologic hallmarks of Alzheimer disease (AD): amyloid-β (Aβ) plaques and tau neurofibrillary tangles. Observing their progressive accumulation in the brains of clinically normal older adults is critically important to understand the pathophysiologic cascade leading to AD and to inform the choice of outcome measures in prevention trials.

**Objective:**

To assess the associations among Aβ, tau, and cognition, measured during different observation periods for 7 years.

**Design, Setting, and Participants:**

Prospective cohort study conducted between 2010 and 2017 at the Harvard Aging Brain Study, Boston, Massachusetts. The study enrolled 279 clinically normal participants. An additional 90 individuals were approached but declined the study or did not meet the inclusion criteria. In this report, we analyzed data from 60 participants who had multiple Aβ and tau PET observations available on October 31, 2017.

**Main Outcomes and Measures:**

A median of 3 Pittsburgh compound B–PET (Aβ, 2010-2017) and 2 flortaucipir-PET (tau, 2013-2017) images were collected. We used initial PET and slope data, assessing the rates of change in Aβ and tau, to measure cognitive changes. Cognition was evaluated annually using the Preclinical Alzheimer Cognitive Composite (2010-2017). Annual consensus meetings evaluated progression to mild cognitive impairment.

**Results:**

Of the 60 participants, 35 were women (58%) and 25 were men (42%); median age at inclusion was 73 years (range, 65-85 years). Seventeen participants (28%) exhibited an initial high Aβ burden. An antecedent rise in Aβ was associated with subsequent changes in tau (1.07 flortaucipir standardized uptake value ratios [SUVr]/PiB-SUVr; 95% CI, 0.13-3.46; *P* = .02). Tau changes were associated with cognitive changes (−3.28 *z* scores/SUVR; 95% CI, −6.67 to −0.91; *P* = .001), covarying baseline Aβ and tau. Tau changes were greater in the participants who progressed to mild cognitive impairment (n = 6) than in those who did not (n = 11; 0.05 SUVr per year; 95% CI, 0.03-0.07; *P* = .001). A serial mediation model demonstrated that the association between initial Aβ and final cognition, measured 7 years later, was mediated by successive changes in Aβ and tau.

**Conclusions and Relevance:**

We identified sequential changes in normal older adults, from Aβ to tau to cognition, after which the participants with high Aβ with greater tau increase met clinical criteria for mild cognitive impairment. These findings highlight the importance of repeated tau-PET observations to track disease progression and the importance of repeated amyloid-PET observations to detect the earliest AD pathologic changes.

## Key Points

**Question:**

Is cognitive decline associated with amyloid-β or tau tangles accumulation?

**Findings:**

In this cohort study that included 60 normal older adults with repeated positron emission tomography measures, the rate of tau accumulation in the inferior temporal neocortex was associated with the rate of cognitive decline. Amyloid accumulation was associated with subsequent tau accumulation, and this sequence of successive amyloid and tau changes in neocortex was found to mediate the association of initial amyloid with final cognition, measured 7 years later.

**Meaning:**

Amyloid positron emission tomography is useful to detect early Alzheimer pathology; repeated tau positron emission tomography is useful to track disease progression.

## Teaser

This cohort study assesses the associations among amyloid-β, tau, and cognition, measured during different observation periods for 7 years.

## Introduction

Alzheimer disease (AD) is a progressive cognitive disorder leading to dementia^1^ in which the brain gradually accumulates both amyloid-β (Aβ) and tau pathologies.^2^ Autopsy studies identified the early stages of Aβ and tau pathologies in individuals who were clinically normal during life, representing a preclinical stage of AD.^3^ Based on autopsy studies,^4^ the prevailing research hypothesis posits that Aβ precedes and accelerates neocortical tau pathology, which together precipitate cognitive decline. Molecular positron emission tomography (PET) tracers for Aβ^5^ and tau^6^ have made it possible to detect these pathologies in living individuals, including in clinically normal adults. However, previous longitudinal PET studies tracked either Aβ^7,8^ or tau^9,10^ accumulation; to our knowledge, a temporal sequence of Aβ and tau accumulation has not yet been evaluated. To observe the sequence of these pathologic events, we investigated the trajectories and temporal courses of longitudinal Aβ-PET and longitudinal tau-PET data. Improved understanding of these trajectories is needed to efficiently test therapeutic strategies designed to halt the progression of pathology and delay cognitive decline. While cognitive decline has been demonstrated in longitudinal studies of older adults with elevated levels of Aβ pathology at baseline,^7,11,12,13^ tau-PET may be more closely linked to neuronal injury and cognition.^14,15,16^ We therefore conducted a prospective natural history study to determine whether serial Aβ and tau measures were associated with concurrent and subsequent, serial measures of cognitive performance.

## Methods

### Participants

In this report, we analyzed data from the Harvard Aging Brain study, a longitudinal study of aging conducted at Massachusetts General Hospital, Boston. We reported prospective observations collected from January 1, 2010, to October 31, 2017, from 60 individuals who had normal cognition at study entry: global Clinical Dementia Rating of 0 and/or Mini Mental State Examination (MMSE) and Wechsler Logical Memory II delayed recall (LM) scores within normal range (MMSE ≥27 and LM ≥11 if ≥16 years of education and MMSE ≥25 and LM ≥7 otherwise). Exclusion criteria included drug or alcohol abuse, head trauma, and serious medical or psychiatric condition (Geriatric Depression Scale >10 of 30). Annual consensus meetings evaluated progression to mild cognitive impairment (MCI).^17^ The Partners Institutional Review Board has approved the Harvard Aging Brain study protocol, and participants provided written informed consent before undergoing any procedures. The participants analyzed in this report had multiple flortaucipir (FTP, also known as AV1451 or T807) and Pittsburgh compound B (PiB) PETs assessing tau and Aβ pathology, respectively.

### Study Design

**Figure 1.**
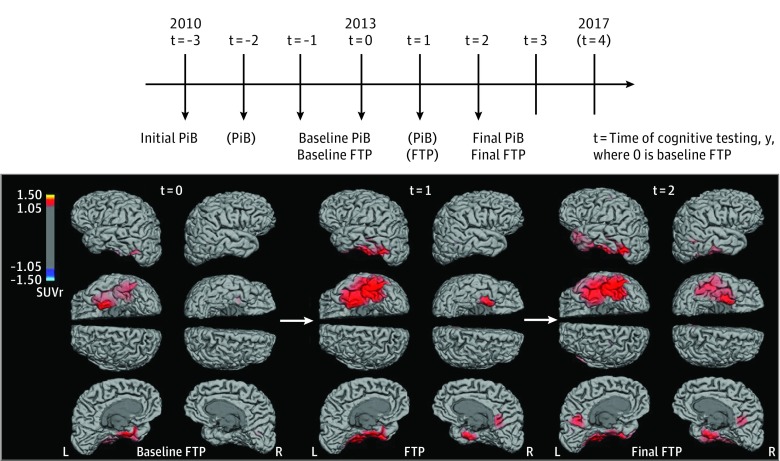
Research Design and Serial Tau–Positron Emission Tomography (PET) Surface Images of an Illustrative Participant A, Baseline of this study was defined as the time of baseline FTP imaging (*t* = 0; where *t *indicates time in years from baseline). Change in flortaucipir (FTP) was evaluated between *t* = 0 and *t* = 2. Pittsburgh compound B (PiB) and cognitive data have been evaluated both between *t* = −3 and *t* = 0 and between *t* = 0 and *t* = 3. Parentheses mean that less than half the sample was observed. The brain images are of an illustrative participant with high PiB at baseline FTP-PET (ε4 noncarrier). Global Clinical Dementia Rating remained stable at 0 during the follow-up, but Preclinical Alzheimer Cognitive Composite *z* scores declined from 0.01 (*t* = 0) to −0.88 (*t* = 3). Note the progressive extension of FTP-PET signal from left entorhinal cortex to left temporal neocortex, posterior cingulate, and to the homologous regions in the right hemisphere. The FTP-PET images use a threshold set at standardized uptake value ratios (SUVr) of 1.05, with cerebral white matter as reference and partial volume correction.

Longitudinal data (Figure 1) were acquired for PiB and cognition from 2010. Because FTP was not available before 2013, the initial FTP was defined as baseline (time*_t_* _= 0_, where subscript *t* indicates time in years), and the terms baseline FTP*_t_* _= 0_ and initial FTP*_t_* _= 0_ are equivalent. The initial PiB was acquired a median of 3 years before baseline and is termed *initial PiB_t_ _= -3_*, with *baseline PiB_t_ _= 0_* referring to PiB at approximately the time of baseline FTP*_t_*   _= 0_, with a median time difference of −1.1 months (range, −9.7 to 9.0).

Final PiB*_t_* _= 2_ was performed at the same time as final FTP *_t_* _= 2_, with a median time difference of 0.0 months (range, −6.3 to 23.4). Baseline cognition*_t_* _= 0_ was evaluated within 6 months of baseline FTP*_t_* _= 0_, with a median time difference of −1.2 months (range, −5.9 to 5.5). Final cogniton*_t_* _= 3_ was evaluated 11.8 months (range, −3.9 to 26.2) after final FTP*_t_* _= 2_.

Participants had 2 or 3 FTP observations (n = 9) over a median follow-up of 26.0 months (range, 13.1-36.4). Participants had 2 to 5 PiB and 4 to 8 cognitive sessions. Pittsburgh compound B and cognition were measured in 2 successive periods: before and after baseline FTP*_t_* _= 0._ Pittsburgh compound B changes were measured before baseline for 36.4 months (range, 15.9-63.5) and after baseline for 24.4 months (range, 16.6-49.3). Cognitive changes were measured before baseline for 34.6 months (range, 4.3-48.8) and after baseline for 38.2 months (range, 23.2-50.5). In 10 participants without prebaseline PiB data, PiB change was assessed from PiB*_t_* _= 0_.

### Neuropsychologic Evaluation

Participants in the Harvard Aging Brain study are evaluated annually with a battery of cognitive assessments, including tests of episodic memory, executive function, global cognition, and the Clinical Dementia Rating. For this study, we evaluated cognition using the Preclinical Alzheimer Cognitive Composite (PACC-96), a mean of *z* score performances on 4 tests sensitive to cognitive decline in at-risk individuals: MMSE, LM, Digit-Symbol Coding, and the Free and Cued Selective Reminding Test, which uses 3 versions with different items, each version repeated every 3 years.^12^

### Molecular Imaging

The ^11^C-PiB and F-18–FTP tracers were synthesized and administered onsite. Positron emission tomography images were acquired using a Siemens HR+ scanner. Both PiB and FTP measures were computed as standardized uptake value ratios (SUVr; 4 frames of 5 minutes: 80-100 minutes for FTP and 40-60 minutes for PiB) using cerebral white matter as the reference region^8,18,19^ because this reference provided more stable estimates of both PiB and FTP change^10^ (eAppendix in the Supplement).^20^ Positron emission tomography data were coregistered to each participant’s magnetic resonance imaging and segmented with Freesurfer, version 5 (Martinos Center for Biomedical Imaging). For each participant, we selected the magnetic resonance imaging closest to the midpoint between FTP sessions.^10^ Partial volume correction was applied using geometric transfer matrix.^16^ The PiB signal was extracted from a neocortical aggregate^21^ and FTP from inferior temporal, a region where tau is commonly observed in preclinical AD.^6^ Additional brain regions were investigated in eTables 1 and 2 in the Supplement; similar results were observed in the temporal neocortex and precuneus. High-PiB threshold was set at 0.724 SUVr using a Gaussian mixture model on the initial PiB*_t_* _= -3_ data.^21^ Some analyses focused on participants with low PiB to evaluate the contribution of subthreshold PET signal accumulation.

### Statistics

**Figure 2.**
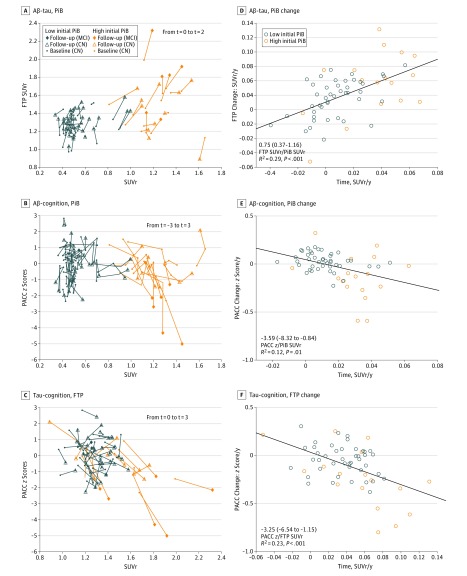
Longitudinal Associations Between Amyloid-β (Aβ), Tau, and Cognition, Observed Contemporaneously A-C, Spaghetti plots showing the unadjusted positron emission tomography (PET) standardized uptake value ratios (SUVr) and Preclinical Alzheimer Cognitive Composite (PACC) scores at the initial *t* = −3 (n = 50; where *t *indicates time in years from baseline), baseline *t* = 0 (n = 60), and follow-up *t* = 2 (N = 60) observations. All MCI progressors had high Pittsburgh compound B (PiB) signal; they were not different than other participants with high PiB at baseline (similar age, PACC, PiB, and FTP), and their PiB change was not particularly fast (B, vertical orange lines ending with a star). However, they had fast FTP and PACC changes (C plot, oblique orange lines). D-F, PiB, FTP, and PACC slope data observed simultaneously are plotted against each other. All associations are significant, although the PiB-PACC longitudinal association is weaker than the PiB-FTP or the FTP-PACC association (Table 2; model 7), probably reflecting that PiB and PACC changes are more distant in time than PiB and FTP or FTP and PACC changes.

**Figure 3.**
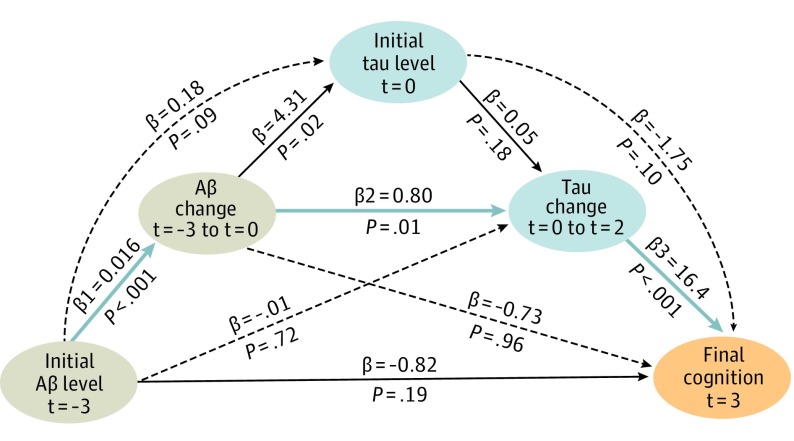
Overview of Sequential Associations Between Amyloid-β (Aβ), Tau, and Cognition Diagram of mediation model pathways relating Aβ, tau, and cognition. Each observation was measured at different, successive times. The mediation highlighted in blue (indirect effect: −0.21; 95% CI, −0.55 to −0.06; *P* = .06) accounts for 20% of the direct effect between initial Pittsburgh compound B (PiB) and final Preclinical Alzheimer Cognitive Composite (PACC) *t* = 3, where *t *indicates time in years from baseline. Altogether, the pathways explain 45% of the direct effect. Black dotted lines illustrate alternative pathways that were not significant. This serial mediation supports a temporal sequence of phenomena in preclinical Alzheimer disease. It is consistent with Table 2, models 1, 4, and 7. It is associated with final PACC *t* = 3 (not PACC slope as in model 7) to dissociate the time of the outcome measure from the time of the predictors measure. Sixty participants were included in this analysis, using baseline PiB *t* = 0 instead of initial PiB *t* = −3 for the 10 participants missing the initial PiB observation. Highly similar results were obtained when excluding these 10 participants.

We computed linear mixed models with random intercept and time slope per participant predicting PACC, FTP, and PiB over time, in separate models. Individual slopes of change were calculated by summing the estimated fixed and random effects of time. For PiB and PACC data, slopes were estimated for the entire follow-up (PiB*_t_* _= -3_ to PiB*_t_* _= 2_), and for shorter periods (referred to as PiB*_t_* _= -3_ to PiB*_t_* _= 0_ before baseline and PiB*_t_* _= 0_ to PiB*_t_* _= 2_ after baseline). Cross-sectional measures and slope data were entered as predictors or outcomes in linear regressions evaluating the associations between PACC, FTP, and PiB and their respective slopes. Five thousand–iteration bootstrapping procedures that accounted for the 2-stage estimation procedure generated 95% confidence intervals. Older ages were associated with steeper PACC slope but not with greater FTP or PiB slope (eFigure 1 in the Supplement); all models predicting PACC slope were therefore adjusted for age. Nine statistical models are displayed in Tables 1 and 2 and another 4 models are displayed in Figures 2 and 3. We did not correct for multiple comparisons.^22,23^ Results were summarized in a serial mediation model (Figure 3), providing evidence for sequential biomarker changes in preclinical AD. This model tested whether the association of initial PiB*_t_* _= -3_ with final PACC*_t_* _= 3_ was mediated by sequential changes in PiB*_t_* _= -3_ to PiB*_t_* _= 0_ and FTP*_t_* _= 0_ to FTP*_t_* _= 2_, adjusting for age, sex, education, and initial PACC*_t_* _= -3._ All possible indirect pathways between PiB, FTP, and final PACC *_t_* _= 3_ scores were tested. Total, direct, and indirect associations were tested using a 5000-iteration bootstrapping procedure.^24^ Models were fit in Matlab, version 9.0 (MathWorks), except mediation models, which used R, version 3.4.2, Lavaan package (the R Foundation). We report 2-sided *P *values with a significance of .05.

**Table 1.** Characteristics of the Participants^a^

Value	Mean (SD)			95% CI	*P* Value
	All (N = 60)	Low PiB (n = 43)	High PiB (n = 17)		
Age at inclusion, *t* = −3, y	73.1 (6.0)	72.6 (6.1)	74.4 (5.5)	−1.6 to 5.2	.29
Education, y	15.6 (3.2)	15.4 (3.4)	16.1 (2.7)	(−1.2 to 2.4	.50
Female, No. (%)	35 (58.3)	24 (55.8)	11 (64.7)	NA	.54
ε4 Carriers, No. (%)	20 (33.9)	8 (19.0)	12 (70.6)	NA	<.001
Missing	1	1	0	NA	NA
Initial PiB: *t* = −3, SUVr	0.66 (0.31)	0.49 (0.09)	1.11 (0.20)	0.53 to 0.68	<.001
Missing	10	8	2		
Baseline PiB: *t* = 0, SUVr	0.71 (0.34)	0.52 (0.13)	1.20 (0.17)	0.59 to 0.77	<.001
Final PiB: *t* = 2, SUVr	0.74 (0.36)	0.53 (0.14)	1.27 (0.16)	0.65 to 0.82	<.001
Annual PiB change					
Period 1: *t* = −3 to *t* = 0	0.01 (0.01)	0.01	0.02 (0.01)	0.01 to 0.02	<.001
95% CI^b^	0.01 to 0.02	0.00 to 0.01	0.02 to 0.03	NA	NA
CoV, SUVr/y	0.8	0.9	0.4	NA	
Missing	10	8	2	NA	
Period 2: *t* = 0 to *t* = 2	0.01 (0.02)	0.01 (0.02)	0.03 (0.03)	0.02 to 0.04	<.001
95% CI^b^	0.01 to 0.02	0.00 to 0.01	0.02 to 0.05	NA	
CoV, SUVr/y	1.6	2.7	0.7	NA	
Baseline FTP: *t* = 0, SUVr	1.29 (0.18)	1.24 (0.12)	1.43 (0.24)	0.10 to 0.28	<.001
Final FTP: *t* = 2, SUVr	1.38 (0.23)	1.31 (0.12)	1.55 (0.34)	0.12 to 0.35	<.001
Annual FTP change					
Period 2: *t* = 0 to *t* = 2	0.04 (0.03)	0.03 (0.03)	0.05 (0.04)	0.001 to 0.04	.04
95% CI^b^	0.03 to 0.05	0.03 to 0.04	0.03 to 0.08	NA	
CoV, SUVr/y	0.8	0.7	0.8	NA	
Initial PACC: *t* = −3, *z* score	−0.06 (0.88)	−0.09 (0.95)	0.00 (0.72)	−0.52 to 0.61	.87
Baseline PACC: *t* = 0, *z* score	0.00 (1.00)	0.10 (1.02)	−0.25 (0.93)	−0.92 to 0.22	.23
Final PACC: *t* = 3, *z* score	−0.31 (1.40)	0.03 (1.06)	−1.18 (1.64)	−1.96 to −0.47	.002
Annual PACC change					
Period 1: *t* = −3 to *t* = 0	0.05 (0.07)	0.06 (0.06)	0.01 (0.08)	−0.09 to −0.01	.02
95% CI^b^	0.03 to 0.07	0.04 to 0.08	−0.03 to 0.08	NA	
CoV, SD/y	1.5	1.0	5.9	NA	
Period 2: *t* = 0 to *t* = 3	−0.10 (0.23)	−0.05 (0.16)	−0.25 (0.31)	−0.32 to −0.08	.002
95% CI^b^	−0.16 to −0.05	−0.10 to −0.00	−0.41 to −0.09	NA	
CoV, SD/y	2.2	3.2	1.3	NA	

Abbreviations: CoV, coefficients of variation; ε4, epsiolon allele; FTP, flortaucipir; NA, not applicable; PACC, Preclinical Alzheimer Cognitive Composite; PET, positron emission tomography; PiB, Pittsburgh compound B; SUVr, standardized uptake value ratios; *t*, time in years from baseline.

^a^ Participants with low and high PiB are compared using *t* tests (χ^2^ for ε4 genotype and sex). The 95% CIs are provided for the difference between PiB groups (last column). The 95% CI within groups are also provided for change data to assess whether they significantly differed from zero. Coefficients of variation (CoV = SD of change divided by mean change) are provided for PET and PACC changes. Change data are slopes extracted from separate linear mixed-effect models measuring PiB, FTP, and PACC over time with a random intercept and time slope per participant.

^b^ *P* < .05.

**Table 2.** Linear Regressions Investigating the Longitudinal Associations Between Amyloid (PiB-PET), Tau (FTP-PET), and Cognition (PACC Performances)^a^

Model No.	Outcome	Factors	Estimate (95% CI)	Two-tailed *P* Value
1^b^	FTP change (*t* = 0 to *t* = 2)	PiB change (*t* = −3 to *t* = 0)	1.13 (0.13 to 3.46)	.02
		Initial PiB (*t* = −3)	0.00 (−0.04 to 0.05)	.80
2	Final PiB (*t* = 2)	FTP change (*t* = 0 to *t* = +2)	1.36 (−2.41 to 6.69)	.44
		Baseline FTP (*t* = 0)	0.89 (0.45 to 1.37)	<.001
3	Final FTP (*t* = 2)	PiB change (*t* = 0 to *t* = +2)	3.64 (0.29 to 6.53)	.03
		Baseline PiB (*t* = 0)	0.17 (−0.06 to 0.48)	.18
4	Final FTP (*t* = 2)	PiB change (*t* = −3 to *t* = +2)	6.87 (2.46 to 12.70)	<.001
		Initial PiB (*t* = −3)	0.12 (−0.13 to 0.51)	.31
5^d^	FTP change (*t* = 0 to *t* = 2)	Baseline PiB (SD) (*t* = 0)	0.001 (−0.008 to 0.016)	.72
		Baseline FTP (SD) (*t* = 0)	0.001 (−0.007 to 0.009)	.80
		Baseline PiB and baseline FTP	0.13 (0.002 to 0.25)	.01
6^d^	PiB change (*t* = 0 to *t* = 2)	Baseline PiB (SD) (*t* = 0)	0.10 (0.003 to 0.19)	.004
		Baseline FTP (SD) (*t* = 0)	0.001 (−0.006 to 0.008)	.88
		Baseline PiB and baseline FTP	0.004 (−0.003 to 0.009)	.33
7^c^	PACC change (*t* = 0 to *t* = 3)	Baseline PiB (SUVr) (*t* = 0)	−0.19 (−0.44 to −0.003)	.05
		PiB change (SUVr/y) (*t* = 0 to *t* = +2)	1.75 (−1.44 to 5.36)	.31
		Baseline FTP (SUVr) *t* = 0)	−0.17 (−0.69 to 0.18)	.40
		FTP change (SUVr/y) (*t* = 0 to *t* = +2)	−3.28 (−6.67 to −0.91)	.001
8^c^^,^^d^	PACC change (*t* = 0 to *t* = 3)	Baseline PiB (SUVr) (*t* = 0)	−0.03 (−0.11 to 0.07)	.32
		PiB change (SUVr/y) (*t* = 0 to *t* = +2)	2.08 (−1.08 to 5.50)	.21
		Baseline FTP (SUVr) (*t* = 0)	0.01 (−0.50 to 0.36)	.94
		FTP change (SUVr/y) (*t* = 0 to *t* = +2)	−2.62 (−6.31 to −0.40)	.01
		Baseline PiB (SD) and FTP change	−1.38 (−3.18 to −0.05)	.04
9^c^	PACC change (*t* = 2 to *t* = 3)	FTP change (*t* = 0 to *t* = +2)	−8.59 (−17.51 to 0.33)	.05
		Final FTP (*t* = +2)	0.38 (−1.01 to 1.76)	.58
		Baseline PACC (*t* = 0)	0.12 (−0.10 to 0.34)	.28

Abbreviations: FTP, Flortaucipir; PACC, Preclinical Alzheimer Cognitive Composite; PET, positron emission tomography; PiB, Pittsburgh compound B; SUVr, standardized uptake value ratios; *t*, time in years from baseline.

^a^ Unadjusted estimates between PiB and FTP changes are provided with 95% confidence intervals generated from a 5000-iteration bootstrap; N = 60.

^b^ Model 1 only includes the 50 participants with PiB data preceding baseline FTP *t* = 0.

^c^ Models 7-9 are adjusted for baseline age, sex, and education, which are not significant (not shown).

^d^ Baseline PiB and FTP SUVr data have been *z *scored in models 5, 6, and 8 and are thus expressed in SD. This was done to facilitate the interpretation of the main effects: the FTP main effect is given at the mean PiB value (0.0 PiB SD). Interactions between other factors (PiB change and FTP change, baseline FTP and PiB change, or baseline PiB and PiB change) were not significant when FTP change was entered in the model.

## Results

### Characteristics of the Participants During the Study

Demographics, cognitive, and PET data of the 60 participants are provided in Table 1. Based on the initial PiB*_t_* _= -3_, 43 participants were classified as low PiB and 17 as high PiB. During the first observation period of the 3 years prior to baseline FTP, PiB increased and 3 participants progressed from low PiB to high PiB. The PACC scores also increased, presumably indicating practice effects. Participants with high PiB had faster PiB increase and lower practice effect than participants with low PiB. After the first period, no participant met the criteria for MCI.

During the second observation period from baseline to 3 years after baseline, PiB increased, FTP increased, and PACC decreased. Although all these changes were greater than 0 in participants with low PiB (Table 1), participants with high PiB had faster PiB increase, FTP increase, and PACC decline. Six participants with high PiB and no participants with low PiB met clinical criteria for MCI at study end.

### Associations Between Aβ-PET and Tau-PET

We first observed that FTP*_t_* _= 0_ to FTP*_t_* _= 2_ changes were associated with contemporaneous PiB*_t_* _= 0_ to PiB*_t_* _= 2_ changes (Figure 2A and D), indicating a longitudinal association between AD pathologies. We then observed that an early PiB*_t_* _= -3_ to PiB*_t_* _= 0_ rise was associated with later FTP*_t_* _= 0_ to FTP*_t_* _= 2_ changes, regardless of initial PiB*_t_* _= -3_ (Table 2; model 1). However, we could not evaluate whether an early FTP rise was associated with later PiB changes because FTP was not measured in the first observation period. Therefore, we investigated whether FTP or PiB changes in the second observation period were associated with final PiB or FTP, respectively. Consistent with PiB increases preceding FTP, we observed that previous FTP*_t_* _= 0_ to FTP*_t_* _= 2_ changes were not associated with later PiB*_t_* _= 2_ (model 2), but previous PiB*_t_* _= 0_ to PiB*_t_* _= 2_ changes were associated with later FTP*_t_* _= 2_ (model 3). When measured over a longer period preceding the FTP measure, PiB changes were even more closely associated with final FTP*_t_*_= 2_ levels (model 4).

To investigate whether baseline PiB and FTP were independently or synergistically associated with subsequent changes in PiB and FTP, we investigated their main and interactive effects. We observed that FTP*_t_* _= 0_ to FTP*_t_* _= 2_ change was associated with the interaction between baseline PiB*_t_* _= 0_ and FTP*_t_* _= 0_ (model 5), but PiB*_t_* _= 0_ to PiB*_t_* _= 2_ change was only associated with baseline PiB*_t_* _= 0_ (model 6), providing evidence that PiB changes occurred independently of baseline FTP levels, while FTP changes were contingent on baseline PiB levels.

### Associations Between Aβ-PET, Tau-PET, and Cognition

We next aimed to investigate a hypothetical sequence between PiB, FTP, and PACC. The PiB*_t_* _= 0_ was not significantly associated with PACC*_t_* _= 0_ at the cross-section, but FTP*_t_* _= 0_ was (−1.50; 95% CI, −2.90 to −0.10; *P* = .04). The PACC was more closely associated with FTP than with PiB at all times when PiB and FTP competed in the same models, suggesting that FTP signal is more proximal to PACC decline than PiB signal. Similarly, PiB slope and PACC slope were not significantly associated in both periods of observations. The PiB slope was associated with PACC slope for the entire study (Figure 2B and E), but this association did not survive adjusting for initial PiB*_t_* _= -3_. In contrast, FTP slope was associated with PACC slope (Figure 2C and F), including after covarying FTP*_t_* _= 0_.

In a multiple regression estimating PACC*_t_* _= 0_ to PACC*_t_* _= 3_ change with baseline and change in PiB*_t_* _= 0_ to PiB*_t_* _= 2_ and FTP*_t_* _= 0_ to FTP*_t_* _= 2_, only baseline PiB*_t_* _= 0_ and FTP*_t_* _= 0_ to FTP*_t_* _= 2_ change were significant (Table 2; model 7). The interaction between PiB*_t_* _= 0_ and FTP*_t_* _= 0_ to FTP*_t_* _= 2_ change was also significant (model 8), such that FTP change had a greater association with PACC decline at higher PiB-SUVr. Remarkably, although the association of FTP change with PACC change was greater in high PiB, it was also marginally present in individuals with low PiB (−2.39; 95% CI, −6.05 to 0.29; *P* = .09).

Flortaucipir change was associated with clinical progression from preclinical to prodromal AD.^17^ Despite the small sample size, the participants with high PiB who progressed to MCI (n = 6) had significantly greater FTP*_t_* _= 0_ to FTP*_t_* _= 2_ change (0.05 SUVr per year; 95% CI, 0.03-0.07; *P* = .001, eFigure 2 in the Supplement) than the stable participants with high PiB (n = 11). The stable participants with high PiB had similar FTP*_t_* _= 0_ to FTP*_t_* _= 2_ change compared with the participants with low PiB (n = 43), highlighting that only a subgroup of participants with high PiB had fast FTP increase, ie, those who progressed to MCI. The PiB change did not differ between those who progressed to MCI and stable participants with high PiB.

### Sequential Mediation Between Aβ-PET, Tau-PET, and Cognition 

The previous models pointed to FTP change as the strongest factor associated with PACC change, potentially because FTP change was closer in time to PACC change. Because FTP change was associated with previous PiB*_t_* _= -3_ to PiB*_t_* _= 0_ change (Table 2; model 1), we inquired whether a sequence of successive change in PiB and FTP could account for the association between initial PiB*_t_* _= -3_ and final PACC*_t_* _= 3_ scores (PACC SD, −1.50 per PiB-SUVr; *P* = .004). To this end, we modeled a serial mediation assessing different possible pathways between Aβ, tau, and cognition.^24^ This model demonstrated that initial PiB*_t_* _= -3_ was associated with sequential changes, first in PiB*_t_* _= -3_ to PiB*_t_* _= 0_, and then in FTP*_t_* _= 0_ to FTP*_t_* _= 2_, and this sequence was associated with final PACC *_t_* _= +3_ scores (Figure 3). After mediation, the direct association of initial PiB*_t_* _= -3_ with final PACC*_t_* _= 3_ became nonsignificant because it reduced from −1.50 to −0.82 (45%).

### Implications for Clinical Trials

Our results raise the possibility that halting tau accumulation would prevent cognitive decline. To evaluate the potential advantage of using serial FTP-PET measures in trials, we directly compared the association of FTP*_t_* _= 0_ to FTP*_t_* _= 2_ change and final FTP*_t_* _= 2_ with PACC*_t_* _= 2_ to PACC*_t_* _= 3_ change observed after the final FTP*_t_* _= 2_ measure. We found that cognitive decline had a greater association with the longitudinal measure of FTP change than with the cross-sectional measure of FTP (Table 2; model 9), indicating that trials would benefit from serial FTP-PET measures to better identify participants at risk of subsequent decline.

## Discussion

In this prospective study, we followed up clinically normal older adults in the preclinical phase of AD for a period of 7 years and observed an antecedent rise in Aβ to be associated with subsequent tau accumulation in inferior temporal cortex. We found this sequence to be strongly associated with cognitive decline. All participants were clinically normal at baseline, but the subgroup of individuals with high Aβ with fast tau increase met clinical criteria for MCI at follow-up.^17^ Our results indicate a sequence of observable phenomena in preclinical AD:

1. Amyloid-β increase was the initial event observed, including in those with low-Aβ levels. In the first observation period, Aβ increased but cognition did not decline until the second period. Tau measures were not available in the first period, but Aβ measures were associated with subsequent tau changes (model 1) and final tau levels (models 3-5). Two studies^25,26^ also found that an antecedent rise in Aβ was associated with final tau regardless of Aβ levels, but they had no longitudinal tau-PET data to investigate sequential changes.
2. Tau increase in inferior temporal neocortex, while measurable in low-Aβ individuals, was faster in those who were increasing Aβ. A longitudinal tau-PET study^9^ observed that tau increased faster in high-Aβ than in low-Aβ clinically normal adults and another did not,^10^ but neither provided longitudinal Aβ-PET data. Our data indicate that tau changes are more closely associated with the rate of Aβ change than by Aβ levels (model 1). A short delay between Aβ and tau increases is likely to occur in some individuals, as suggested by 3 participants initially classified as low PiB who had both PiB and FTP increase after crossing the threshold for PiB-PET positivity (Figure 2B and C).
3. Cognitive decline was most closely associated with tau change, beyond baseline Aβ and tau. Model 9 indicated that tau changes were associated with subsequent cognitive changes beyond the final FTP scan.
4. After 7 years of cognitive follow-up, criteria for MCI owing to AD were met in a subset of 6 participants with high PiB (35%). These observations, suggesting higher rates of tau accumulation with clinical progression, are consistent with previous studies showing higher rates of tau accumulation in patients with symptomatic AD than in clinically normal older adults.^9,27^ The sequence from subthreshold Aβ accumulation to MCI was not observed in any participant, suggesting it requires longer than 7 years. We did not observe any MCI owing to non-AD etiologies.

Altogether, our findings indicate that Aβ-PET measures have a delayed and indirect, tau-mediated association with cognition. Previous longitudinal PiB data estimated that the threshold for Aβ positivity was reached many years before dementia onset.^7^ We observed that tau changed shortly after Aβ positivity; however, we also observed high variability in tau change among individuals with high Aβ; those with rates of tau change similar to the low-Aβ group had stable cognition, highlighting that the Aβ-cognition delay may be variable and emphasizing the value of measuring tau to track disease progression in preclinical AD.

Cerebrospinal fluid studies also found that longitudinal Aβ and tau trajectories were associated,^28^ and the rate of tau changes, not the rate of Aβ changes, were associated with cognition,^29^ but sequential associations in different times were not investigated using cerebrospinal fluid. Both PET and cerebrospinal fluid data indicate that synergy between Aβ and tau is associated with brain dysfunction,^16,30^ atrophy,^14,31,32^ and cognitive decline.^33,34^ We observed that Aβ and tau in inferior temporal neocortex interacted and potentiated tauopathy (model 5) and cognitive decline (model 8).

The low-Aβ group demonstrated tau increase (Table 1) and an association between tau increase and cognition, albeit weak. In contrast, tau increase was not observed in participants with low PiB in a tau-PET study with a 14-month follow-up.^9^ Our findings indicate that some individuals classified as low Aβ may be on the same trajectory of tau-mediated memory decline as those with high Aβ.^26,35^ Autopsy studies indicate that PiB is not sensitive to prefibrillar or low levels of fibrillar Aβ, which may be biologically active.^36^ The observation that the subthreshold Aβ accumulation was associated with subsequent tau accumulation highlights the limits of cross-sectional Aβ-PET and advocates for using repeated PET measures to improve characterization of preclinical AD.

Our results may inform prevention therapeutic trials. Tau measures showed greater rates and consistency of accumulation than measures of Aβ or cognition (Table 1). Tau-PET outcomes may thus permit more rapid assessment of pharmacodynamic effects and thereby facilitate early phase proof-of-concept trials.^37^ Furthermore, serial tau-PET measures could identify individuals at risk of rapid cognitive decline (Table 2; model 9). Probably because Aβ increases long before cognition declines, Aβ changes did not add information compared with a baseline Aβ-PET to predict cognition; however, Aβ changes were associated with tau changes, suggesting that the effect of Aβ accumulation could be more easily assessed with tau-PET outcomes than with cognitive measures. Lastly, because clinical progression is more closely associated with tau than with Aβ, drugs effectively reducing tau increase on a tau-PET outcome may be more likely to slow down the rate of decline when tested with clinical outcomes.

### Limitations

We did not observe tau at study start because the FTP tracer was not yet available. Although we provided evidence in favor of Aβ preceding inferior temporal tau, we could not test the absence of association between early tau changes and later Aβ changes. Because our data set only included participants older than 65 years, we focused the current work on neocortical tau, but future studies will also need to focus on tau accumulation in the medial temporal lobe, a region in which tau may accumulate at younger ages and may precede Aβ accumulation according to autopsy studies.^38^ Future research will also determine the trajectory of structural and functional neurodegenerative markers respective to changes in Aβ, tau, and cognition as well as their spatial overlaps. A 2019 study^10^ suggested that tau accumulation and brain atrophy share a similar topography. Finally, the relatively modest sample size of this study prevents generalization. Some observations were based on a few individuals with high Aβ who progressed to MCI; we observed an association between tau change and cognitive change in the participants with low Aβ as well, but it was only trend level. All cases may not follow the same temporal progression, and larger studies are thus required to evaluate interindividual variations in biomarkers trajectories.

## Conclusions

In this longitudinal PET study, we observed that successive changes in Aβ and then tau were associated with lower cognition after a 7-year follow-up. Larger samples are needed to validate the proposed sequence. Additional observations will help estimate the delay separating the trajectories of Aβ, tau, and cognition.

## Notes

Supplement. **eAppendix.** PET data processing **eTable 1. **Associations Between PiB Change and FTP Change in Additional ROIs **eTable 2. **Associations Between PACC Change and FTP Change in Additional ROIs **eFigure 1. **Longitudinal Changes in Aβ, Tau, and Cognition With Age **eFigure 2. **Whole-Brain FTP t-Maps Comparing High-PiB CN-to-MCI Progressors and High-PiB Stable CN Participants
