# Supplementary material for: Association of Amyloid and Tau With Cognition in Preclinical Alzheimer Disease: A Longitudinal Study
Source: JAMA Neurol. 2019 Jun 3;76(8):915–24. doi: 10.1001/jamaneurol.2019.1424 (PMC6547132; doi:10.1001/jamaneurol.2019.1424)
Supplement: Supplement. — eAppendix. PET data processing eTable 1. Associations Between PiB Change and FTP Change in Additional ROIs eTable 2. Associations Between PACC Change and FTP Change in Additional ROIs eFigure 1. Longitudinal Changes in Aβ, Tau, and Cognition With Age eFigure 2. Whole-Brain FTP t-Maps Comparing High-PiB CN-to-MCI Progressors and High-PiB Stable CN Participants [file jamaneurol-76-915-s001.pdf]

Supplementary Online Content

Hanseeuw BJ, Betensky RA, Jacobs HIL, et al. Association of amyloid and tau with cognition in preclinical Alzheimer disease: a longitudinal study. *JAMA Neurol*. Published online June 3, 2019. doi:10.1001/jamaneurol.2019.1424

eAppendix. PET data processing
eTable 1. Associations Between PiB Change and FTP Change in Additional ROIs
eTable 2. Associations Between PACC Change and FTP Change in Additional ROIs
eFigure 1. Longitudinal Changes in Aβ, Tau, and Cognition With Age
eFigure 2. Whole-Brain FTP t-Maps Comparing High-PiB CN-to-MCI Progressors and High-PiB Stable CN Participants

This supplementary material has been provided by the authors to give readers additional information about their work.

© 2019 Hanseeuw BJ et al. *JAMA Neurology.*

eAppendix: PET data processing

PET data presented in this work were processed using a specific longitudinal pipeline. The mean PET images (average Counts Per Second - CPS) from each session were realigned to each other in two steps. A first pass aligned follow-up scan(s) to the baseline scan, then an average image was created and a second pass aligned all scans to the mean image. The mean image was used for co-registration to the MRI closest to the midpoint between FTP session date sfor a subject, allowing a single registration to be done. ROIs from the selected MR were then applied to all time points. The aim of this longitudinal pipeline was to try to maximize the registration between time points, as PET to PET should produce a better fit than a set of PET to MR registrations, and should reduce variability.

The choice of cerebral white matter as a reference region has been well documented for longitudinal PET studies using Aβ tracers.[1-4] The first longitudinal tau-PET studies used different reference regions[3] including cerebellar gray[5] and subcortical white matter[6]. In our dataset (n=60), the Pearson's correlation between baseline FTP data scaled on cerebellar gray or scaled on cerebral white matter was 0.85. The correlation between FTP slope data using either reference was 0.71. The present study also used partial volume correction (PVC). The Pearson's correlation between baseline FTP data with and without PVC was 0.87. The correlation between FTP slope data obtained with and without PVC was 0.80 (cerebral white matter reference region used for both PVC and

© 2019 Hanseeuw BJ et al. *JAMA Neurology.*

non-PVC data). In comparison, PVC and the choice of the reference region had smaller impact on PiB cross-sectional and slope data (all R>0.93). We observed associations between PiB slope and FTP slope, and between FTP slope and PACC slope, regardless of PET data processing.

The annual rates of FTP changes were higher in this study than in the recently published longitudinal FTP data from Mayo Clinic.[5] Both studies observed faster rates of FTP change in high-PiB than in low-PiB participants, but the low-PiB participants from the Mayo Clinic did not demonstrate significant FTP changes over a 14-month follow-up. Longer follow-up time (e.g., 26 months in our study) may more readily reveal early tau changes. FTP reference region may also influence measurement consistency: Mayo used cerebellar crus; we used cerebral white matter. Using cerebellar gray as reference, we observed significantly lower rates of FTP change (cerebellar gray: 0.025SUVr/y, cerebral white matter: 0.041SUVr/y, p<0.0001); however, FTP change $_{t=0-2}$ was significantly greater than zero using either reference in the forty participants with low-PiB $_{t=0}$ at baseline (cerebellar gray: 0.021SUVr/y, cerebral white matter: 0.033SUVr/y, one-sample t-tests: both p's<0.0001). Both the Mayo Clinic study and ours used PVC data. The in-press data of Berkeley[6] show rates of FTP accumulation in the inferior temporal neocortex of older adults that are very similar to ours (3% per year in both studies). They also used subcortical white matter as a reference region and PVC.

eTable1: Associations between PiB change and FTP change in additional ROIs

© 2019 Hanseeuw BJ et al. *JAMA Neurology.*

ROI	Outcome	Predictors	Estimate (SE)	2-tailed p
**Inferior temporal**	FTP change (t=0 to t=+2)	PiB change (t=0 to t=+2)	0.78 (0.17)	**<0.001**
		Baseline PiB (t=0)	-0.01 (0.01)	0.66
**Middle temporal**	FTP change (t=0 to t=+2)	PiB change (t=0 to t=+2)	0.30 (0.10)	**0.004**
		Baseline PiB (t=0)	0.01 (0.01)	0.39
**Superior temporal**	FTP change (t=0 to t=+2)	PiB change (t=0 to t=+2)	0.09 (0.05)	**0.05**
		Baseline PiB (t=0)	0.01 (0.003)	0.06
**Isthmus Cingulate**	FTP change (t=0 to t=+2)	PiB change (t=0 to t=+2)	0.24 (0.10)	**0.02**
		Baseline PiB (t=0)	0.01 (0.01)	0.26
**Precuneus**	FTP change (t=0 to t=+2)	PiB change (t=0 to t=+2)	0.08 (0.03)	**0.004**
		Baseline PiB (t=0)	0.001 (0.001)	0.46
**Fusiform**	FTP change (t=0 to t=+2)	PiB change (t=0 to t=+2)	0.81 (0.19)	**<0.001**
		Baseline PiB (t=0)	0.02 (0.01)	0.26
**Temporal meta-ROI**	FTP change (t=0 to t=+2)	PiB change (t=0 to t=+2)	0.86 (0.18)	**<0.001**
		Baseline PiB (t=0)	0.003 (0.01)	0.81

The temporal meta-ROI is a Freesurfer average between fusiform, inferior temporal, middle temporal, and entorhinal cortex. PiB is measured in a neocortical aggregate (FLR).

eTable2: Associations between PACC change and FTP change in additional ROIs

ROI	Outcome	Predictors	Estimate (SE)	2-tailed p
**Inferior temporal**	PACC change (t=0 to t=+3)	Baseline PiB (t=0)	-0.19 (0.09)	**0.05**
		Baseline FTP (t=0)	-0.17 (0.18)	0.40
		FTP change (t=0 to t=+2)	-3.28 (0.90)	**0.001**
**Middle temporal**	PACC change (t=0 to t=+3)	Baseline PiB (t=0)	-0.16 (0.09)	**0.08**
		Baseline FTP (t=0)	-0.15 (0.21)	0.47
		FTP change	-5.80	**<0.001**

© 2019 Hanseeuw BJ et al. *JAMA Neurology.*

		(t=0 to t=+2)	(1.64)	
**Superior temporal**	PACC change (t=0 to t=+3)	Baseline PiB (t=0)	-0.16 (0.09)	**0.09**
		Baseline FTP (t=0)	-0.11 (0.24)	0.66
		FTP change (t=0 to t=+2)	-6.71 (3.85)	**0.09**
**Isthmus Cingulate**	PACC change (t=0 to t=+3)	Baseline PiB (t=0)	-0.14 (0.09)	**0.03**
		Baseline FTP (t=0)	0.05 (0.21)	0.81
		FTP change (t=0 to t=+2)	-3.00 (1.73)	**0.09**
**Precuneus**	PACC change (t=0 to t=+3)	Baseline PiB (t=0)	-0.17 (0.09)	**0.06**
		Baseline FTP (t=0)	-0.003 (0.21)	0.98
		FTP change (t=0 to t=+2)	-17.60 (6.47)	**0.009**
**Fusiform**	PACC change (t=0 to t=+3)	Baseline PiB (t=0)	-0.11 (0.09)	0.21
		Baseline FTP (t=0)	-0.30 (0.22)	0.17
		FTP change (t=0 to t=+2)	-1.38 (0.79)	**0.09**
**Temporal meta-ROI**	PACC change (t=0 to t=+3)	Baseline PiB (t=0)	-0.17 (0.10)	0.10
		Baseline FTP (t=0)	-0.16 (0.19)	0.40
		FTP change (t=0 to t=+2)	-1.84 (1.02)	**0.08**

All models are co-varied for baseline age, sex, education, and PiB slope (t=0 to t=+2). Covariates are not significant. The temporal meta-ROI is a Freesurfer average between fusiform, inferior temporal, middle temporal, and entorhinal cortex. The model for inferior temporal is also given in the main manuscript (Table 2, #7). PiB is measured in a neocortical aggregate (FLR).

Fig.S1: Longitudinal changes in Aβ, tau, and cognition with age

© 2019 Hanseeuw BJ et al. *JAMA Neurology.*

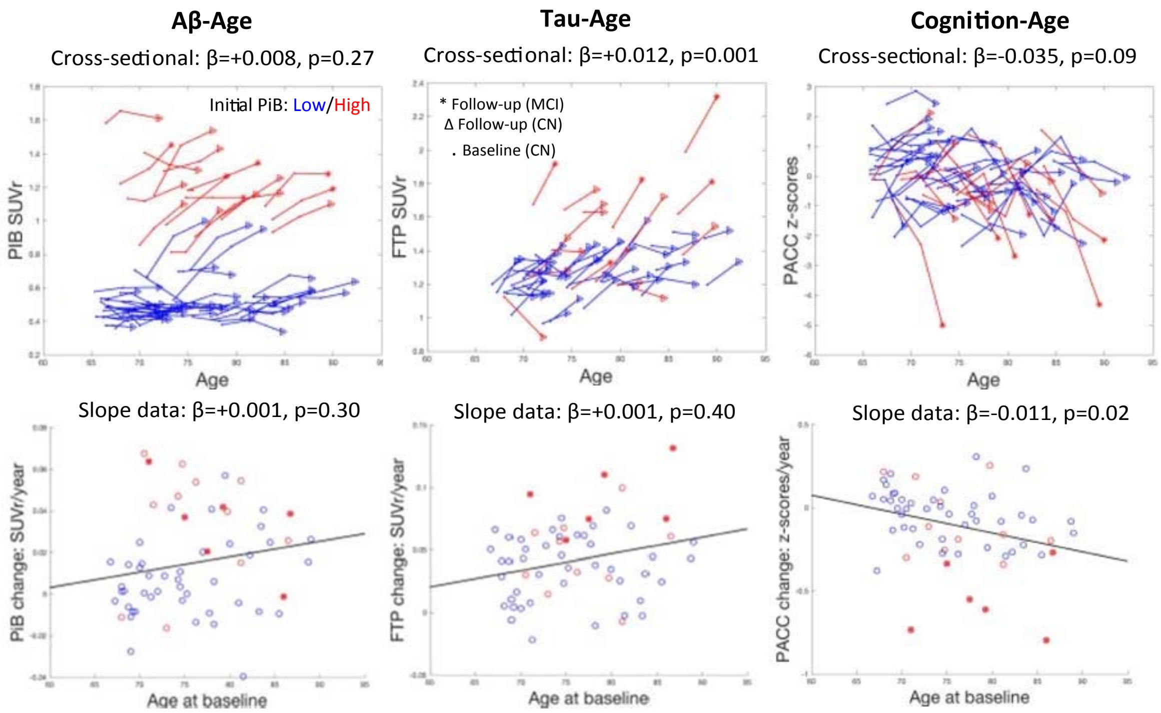

Top: Spaghetti plots showing the unadjusted PET SUVr and PACC scores versus age at the initial$_{t=-3}$, baseline$_{t=0}$, and follow-up$_{t=+2}$ observations. The cross-sectional statistics provided is baseline PACC or PET data predicted by age at baseline. At older ages, PiB signal is not significantly higher, FTP signal is higher, and PACC is marginally lower. Bottom: PiB, FTP, and PACC slope data are plotted against age. Slope data for each outcome was obtained from a linear mixed-model predicting this outcome over time with a random intercept and time slope per subject. The slope data statistics provided is change in PACC (or change in PET data) from baseline to final follow-up predicted by age at baseline, adjusting for baseline PACC (or baseline PET data). PiB and FTP slopes are not associated with age. PACC decline is significantly faster at older ages, adjusting for baseline PACC.

The blue color denotes participants with low-PiB and the red color participants with high-PiB at the initial PiB observation. Initial and baseline data are dots. Follow-up data are triangles for participants who remained clinically normal (CN) during the study and stars for participants who progressed to MCI. All MCI progressors had high-PiB signal.

Fig.S2: Whole-brain FTP t-maps comparing high-PiB CN-to-MCI progressors and high-PiB stable CN participants.

© 2019 Hanseeuw BJ et al. *JAMA Neurology.*

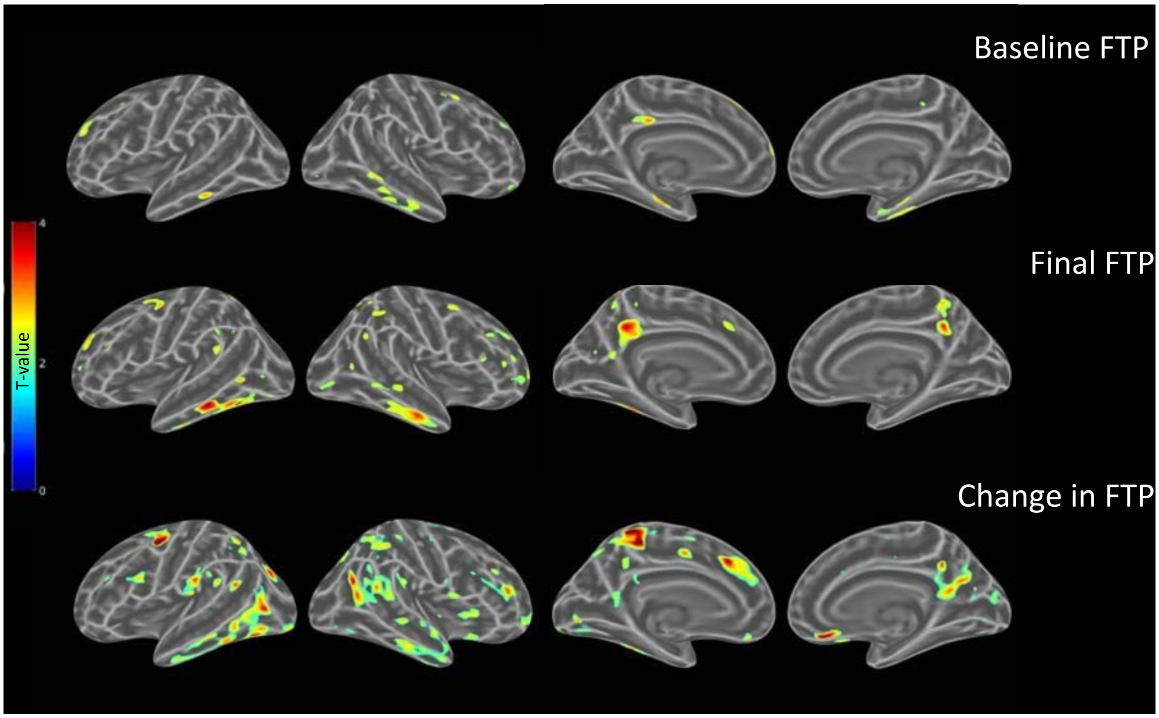

A two-sample t-test compared FTP SUVr in both groups at baseline (top row), final follow-up (middle row), and the SUVr change during the study (bottom row). These maps use a threshold set at t>1.96, two-sided p<0.05. No correction for multiple comparisons was performed as these exploratory analyses were conducted on the small number of high-PIB participants (n=17, including 6 who progressed to MCI and 11 who remained stable). While at baseline, the future MCI progressors did not have higher FTP signal than the other high-PiB participants; FTP signal increased faster in those progressing to MCI, resulting in higher FTP signal in several temporo-parietal regions at final follow-up (after MCI diagnosis).

© 2019 Hanseeuw BJ et al. *JAMA Neurology.*

References:

1. Landau SM, Fero A, Baker SL, et al. Measurement of longitudinal beta-amyloid change with 18F-florbetapir PET and standardized uptake value ratios. *J Nucl Med.* 2015;56(4):567-574.
2. Fleisher AS, Joshi AD, Sundell KL, et al. Use of white matter reference regions for detection of change in florbetapir positron emission tomography from completed phase 3 solanezumab trials. *Alzheimers Dement.* 2017.
3. Southekal S, Devous MD, Sr., Kennedy I, et al. Flortaucipir F 18 Quantitation using a Parametric Estimate of Reference Signal Intensity (PERSI). *J Nucl Med.* 2017.
4. Lowe VJ, Lundt ES, Senjem ML, et al. White matter reference region in PET studies of (11)C-Pittsburgh Compound B uptake: effects of age and amyloid-beta deposition. *J Nucl Med.* 2018.
5. Jack CR, Jr., Wiste HJ, Schwarz CG, et al. Longitudinal tau PET in ageing and Alzheimer's disease. *Brain.* 2018.
6. Harrison TM, La Joie R, Maass A, et al. Longitudinal tau accumulation and atrophy in aging and Alzheimer's disease. *Ann Neurol.* 2018.

© 2019 Hanseeuw BJ et al. *JAMA Neurology.*
